# In-Hospital Complications of Acute Decompensated Heart Failure Patients With Comorbid Rheumatoid Arthritis: A Retrospective Cohort Study

**DOI:** 10.7759/cureus.113417

**Published:** 2026-07-26

**Authors:** Javier Fernandez, Ning Sun, Venkataraghavan Ramamoorthy, Anshul Saxena, Muni Rubens, Sandra Chaparro, Javier Jimenez

**Affiliations:** 1 Department of Medicine, University of Miami Leonard M. Miller School of Medicine, Miami, USA; 2 Miami Cardiac and Vascular Institute, Baptist Health South Florida, Miami, USA

**Keywords:** bleeding risk, heart failure hospitalization, in-hospital outcome, major adverse cardiac effect (mace), rheumatoid arthritis

## Abstract

Introduction

Rheumatoid arthritis (RA) is associated with increased cardiovascular risk and chronic systemic inflammation, which may influence outcomes of heart failure (HF) hospitalization. However, the effect of RA on acute inpatient complications, bleeding risk, and mortality during HF admissions remains unclear.

Methods

We conducted a retrospective cohort study of adults hospitalized with HF across a large tertiary healthcare system from 2016 to 2024. The primary outcome was in-hospital mortality. Secondary outcomes included length of stay, gastrointestinal (GI) and major bleeding, pneumonia, respiratory failure, acute kidney injury (AKI), thromboembolism, and major adverse cardiac events (MACE). Multivariable logistic and negative binomial regression were used to assess associations after adjusting for demographics, comorbidities, laboratory values, and medications. Propensity score matching (PSM) minimized baseline confounding.

Results

The study included 81,366 HF patients. Those with RA were older, more often female, and had higher rates of chronic lung disease, systemic lupus erythematosus (SLE), and thrombocytopenia. After adjustment, RA was associated with higher odds of GI and major bleeding and longer hospital stays, but not with increased mortality or cardiovascular complications. Among those with RA, elevated C-reactive protein was associated with a higher risk of thromboembolic and cardiac complications. Pre-admission disease-modifying antirheumatic drug use was associated with lower thromboembolic risk, but higher risk of major adverse cardiac events.

Conclusion

RA was associated with a greater risk of bleeding complications and prolonged hospitalization during HF admissions but not increased in-hospital mortality. Elevated inflammatory markers and immunosuppressive therapy may contribute to thrombotic and cardiac complications and highlight the need for targeted monitoring strategies during hospitalization.

## Introduction

Heart failure (HF) is a leading cause of hospitalization and mortality worldwide, with a particularly greater burden among patients with chronic inflammatory diseases such as rheumatoid arthritis (RA). RA confers a twofold increased risk of incident HF compared to the general population when controlling for other traditional cardiovascular risk factors and has been associated with ischemic and non-ischemic HF subtypes [1,2]. Mechanistic studies implicate persistent inflammation, immune-mediated myocardial injury, and abnormal cardiac remodeling as critical drivers of HF in RA, with other factors like disease activity and duration further exacerbating risk [3]. These pathophysiological mechanisms represent shared drivers of HF progression across other autoimmune diseases like systemic lupus erythematosus (SLE) and systemic sclerosis [4], as well as post-viral cardiomyopathy, particularly following COVID-19 and in the setting of long COVID [5,6]. In each of these conditions, chronic inflammation promotes microvascular injury and progressive ventricular remodeling, which may predispose to recurrent hospitalizations and HF-related mortality [7]. Despite advances in treatment options for RA, including disease-modifying antirheumatic drugs (DMARDs) and biologic therapies, patients with autoimmune diseases, including RA, experience a higher risk of HF hospitalization and mortality [8,9]. However, the impact of RA on acute inpatient outcomes, such as in-hospital mortality, bleeding, thrombotic complications, and length of stay (LOS), requires ongoing investigations to understand the effects of evolving paradigms and newer medications for managing both HF and RA. Emerging evidence from large retrospective cohorts shows that RA is consistently associated with higher rates of readmission following HF admission; however, mortality risk is unclear with mixed findings [10,11]. These outcomes may be influenced by systemic inflammation and immunosuppressive therapy in RA patients particularly in the acute HF setting. This underscores the need for detailed evaluation of inpatient complications in this high-risk subgroup. 

Furthermore, patients with RA may be at greater risk for bleeding complications through several mechanisms. Chronic nonsteroidal anti-inflammatory drugs (NSAIDs) and steroid use in this population may precipitate direct GI mucosal injury, with a higher likelihood of peptic ulcer formation. Moreover, the presence of extra-articular manifestations in RA patients has been associated with more serious GI bleeding events, which may be related to disease severity contributing to intestinal vulnerability [12]. Endothelial dysfunction driven by chronic inflammation, oxidative stress, and circulating autoantibodies and immune complexes may also compromise vascular integrity and increase susceptibility to bleeding in these patients [13]. Platelets in RA circulate in a chronically activated state that may contribute to hemorrhagic complications via consumptive mechanisms [14]. Lastly, initiation of pharmacologic VTE prophylaxis during HF hospitalization may also significantly compound bleeding risk in these patients.

This study aims to address important gaps in the literature by examining inpatient outcomes and complications among patients hospitalized with HF stratified by RA status. Understanding these associations is essential for improving risk stratification and informing future research on targeted interventions for patients with RA and HF.

This article was previously presented as a meeting abstract at the 2026 International Society of Heart and Lung Transplantation Annual Meeting and Scientific Sessions.

## Materials and methods

Study population

All adults ≥18 years of age admitted to Baptist Health South Florida between January 1, 2016, and May 31, 2024, with a diagnosis of HF (ICD-10-CM:I50.x, I11.0, I13.0, I13.2) were eligible for inclusion. Patients who had missing demographic data were excluded. RA status was ascertained using ICD-10-CM codes M05.x (seropositive RA) and M06.x (other RA). Subjects were categorized into two groups: HF with RA and HF without RA. Within the HF with RA group, a pre-specified subgroup analysis was conducted to examine the association between pre-admission medication use, CRP levels, and in-hospital outcomes. New York Heart Association (NYHA) function classification and other HF severity scores were not available in the dataset.

Outcomes

All outcomes were obtained using ICD-10-CM diagnosis codes during the index hospitalization. The primary outcome of this study was in-hospital mortality. Secondary outcomes included LOS and in-hospital complications such as major adverse cardiac events (MACE), pneumonia, cardiac mortality, atrial fibrillation, cardiogenic shock, GI bleeding, acute kidney injury (AKI), respiratory failure, sepsis, and venous thromboembolism (VTE). MACE was defined as the occurrence of either myocardial infarction (MI), stroke, or cardiovascular death. Major bleeding was defined as neurological, GI, or genitourinary bleeding events during. VTE was defined as deep vein thrombosis (I82.x) or pulmonary embolism (I26.x) during hospital admission. Cardiac arrest was identified using code I46.x. A completed list of ICD-10-CM codes for all baseline comorbidities and outcomes is provided in Supplementary materials 1 and 2. Pre-admission medications, including disease-modifying antirheumatic drugs (DMARDs) and corticosteroids, were also obtained.

Covariates

To account for baseline health conditions, we adjusted for several co-variates such as age, sex, race/ethnicity, tobacco use, obesity, comorbidity burden (treated as binary variables for hyperlipidemia, hypertension, chronic obstructive pulmonary disease, chronic kidney disease, diabetes, peripheral artery disease, SLE, thrombocytopenia, and coronary artery disease), and pertinent laboratory biomarkers such as white blood cell count (WBC), Troponin I, Pro BNP, hemoglobin, sodium, and lactic acid. In the subgroup analysis, additional variables included reconciled at-home use of DMARDs (both conventional and biologic), corticosteroids, and CRP levels measured closest to the admission time categorized as not measured, normal (≤10 mg/L), or high (CRP >10 mg/L).

Statistical analysis

Descriptive analyses were used to summarize the data with frequencies and percentages for categorical variables and means with standard deviations or medians with interquartile ranges for continuous variables depending on the distribution. Bivariate analyses compared patients having versus not having RA using chi-square tests for categorical variables and t-tests or Wilcoxon rank sum tests for continuous variables as determined by the frequency and distribution of the variables. Missing covariate data were imputed using multiple imputation by chained equations (MICE) with the predictive mean matching (PMM) algorithm. 30 imputed datasets were created to ensure estimation stability.

To balance the effect of demographics and comorbidity factors between groups and minimize confounding by general health status, propensity score matching (PSM) was performed at a three-to-one ratio for the non-RA versus the RA group. Standardized mean differences (SMDs) were calculated to evaluate the balance of covariates post-matching. Multivariable logistic regression models were used to assess primary and secondary outcomes. These models adjusted for covariates, including age, sex, race/ethnicity, comorbidities, admission labs, and pre-admission medication use. For the subgroup analyses focusing on patients with RA, multivariable models adjusted for CRP, conventional DMARDs (cDMARDs), biologic DMARDs (bDMARDs), and corticosteroids; synthetic DMARDs (sDMARDs) were excluded from these models due to the small number of users to achieve model convergence.

Results were presented as adjusted odds ratios (aOR) with 95% confidence intervals (CI). For LOS modeled using negative binomial regression, adjusted rate ratio (aRR) with 95% CI was reported. A two-sided p-value of <0.05 was considered statistically significant. All analyses were performed using R version 4.5.1 (R Foundation for Statistical Computing, Vienna, Austria, https://www.R-project.org/).

## Results

A total of 81,366 patients admitted with a diagnosis of HF were included in our analysis and stratified by RA status (79,626 non-RA and 1,740 RA). Baseline patient demographics, characteristics, and in-hospital complications are outlined in Table 1. Patients with RA were older, more likely to be female, and of Hispanic ethnicity. RA patients had a higher prevalence of chronic obstructive pulmonary disease (COPD), SLE, and TCP in contrast to non-RA patients, who were more likely to have coronary artery disease (CAD), diabetes, and CKD at baseline prior to admission. RA patients had a higher proportion of heart failure with preserved ejection fraction (HFpEF) compared to non-RA patients (56% vs. 46%). HF patients with RA also had significantly higher WBC, higher CRP, and lower hemoglobin levels on admission. In terms of in-hospital complications, RA patients had a higher risk of GI bleeding (1.9% vs. 1.0%, p < 0.001) or all major bleeding complications (6.2% vs. 4.5%, p = 0.004) throughout the hospitalization course. RA patients also had a greater risk of prolonged LOS (median 6.0 vs. 5.0 days, p < 0.001); however, no significant difference in all-cause mortality was found (7.9% vs. 6.9%, p = 0.111). There were no differences in other complications.

**Table 1:** Comparison of baseline patient characteristics and in-hospital outcomes between HF with RA versus HF without RA patients HF: heart failure, RA: rheumatoid arthritis, CAD: coronary artery disease, COPD: chronic obstructive pulmonary disease, CKD: chronic kidney disease, PAD: peripheral artery disease, SLE: systemic lupus erythematosus, TCP: thrombocytopenia, AKI: acute kidney injury, MI/ACS: myocardial infarction/acute coronary syndrome, VTE: virtual terminal emulator, MACE: major adverse cardiovascular event, LOS: length of stay, DMARDs: disease-modifying antirheumatic drugs, cDMARDs: conventional DMARDs, bDMARDs: biologic DMARDs, sDMARDs: synthetic DMARDs, HFpEF: heart failure with preserved ejection fraction, HFrEF: heart failure with reduced ejection fraction

	Non-RA; N = 79,626 (97.9%)	RA; N = 1,740 (2.1%)	p-value	Test statistics
Age (median (IQR))	79.00 (68.00, 87.00)	80.00 (74.00, 86.00)	<0.001	-4.909 (Z)
Sex, n (%)				
Male	40,470 (50.9%)	431 (24.8%)	<0.001	461.843 (χ²)
Female	39,156 (49.1%)	1,309 (75.2%)		
Ethnicity				
Black	7,621 (9.6%)	140 (8.1%)	0.002	15.083 (χ²)
Hispanic	53,054 (66.6%)	1,177 (67.6%)		
White	15,795 (20%)	328 (18.9%)		
Other	3,064 (3.8%)	93 (5.4%)		
Comorbidities				
Hyperlipidemia	47,741 (60.0%)	1,037 (59.6%)	0.763	0.091 (χ²)
CAD	42,556 (53.4%)	810 (46.6%)	<0.001	32.505 (χ²)
Hypertension	73,211 (91.9%)	1,621 (93.2%)	0.065	3.417 (χ²)
COPD	17,155 (21.5%)	421 (24.2%)	0.008	7.066 (χ²)
Diabetes	40,726 (51.1%)	807 (46.4%)	<0.001	15.487 (χ²)
Tobacco use	35,418 (44.5%)	742 (42.6%)	0.127	2.327 (χ²)
CKD	33,245 (41.8%)	620 (35.6%)	<0.001	26.337 (χ²)
Obesity	22,310 (28.0%)	484 (27.8%)	0.852	0.035 (χ²)
PAD	4,610 (5.8%)	105 (6.0%)	0.665	0.187 (χ²)
SLE	625 (0.8%)	79 (4.5%)	<0.001	279.961 (χ²)
TCP	8,188 (10.3%)	222 (12.8%)	0.001	11.260 (χ²)
In-hospital complications				
Pneumonia	1,521 (2.2%)	38 (2.6%)	0.410	0.679 (χ²)
Respiratory failure	4,506 (7.9%)	113 (9.3%)	0.136	2.219 (χ²)
Ventricular arrhythmias	1,676 (2.2%)	31 (1.8%)	0.352	0.866 (χ²)
Sepsis	1,617 (2.3%)	45 (3.0%)	0.105	2.626 (χ²)
Stroke	677 (0.9%)	15 (0.9%)	0.958	0.003 (χ²)
AKI	5,010 (9.6%)	117 (9.8%)	0.463	0.539 (χ²)
GI bleeding	795 (1.0%)	31 (1.9%)	0.001	10.394 (χ²)
Major bleeding	3,038 (4.5%)	90 (6.2%)	0.004	8.483 (χ²)
Cardiogenic shock	1,259 (1.6%)	19 (1.1%)	0.104	2.636 (χ²)
MI/ACS	550 (0.8%)	10 (0.7%)	0.563	0.335 (χ²)
Atrial fibrillation	1,688 (3.7%)	30 (3.0%)	0.256	1.290 (χ²)
Cardiac arrest	1,149 (1.5%)	28 (1.6%)	0.566	0.330 (χ²)
VTE	1,139 (1.5%)	33 (2.0%)	0.106	2.606 (χ²)
MACEs	3,301 (5.0%)	65 (4.4%)	0.396	0.722 (χ²)
All-cause mortality	5,490 (6.9%)	137 (7.9%)	0.111	2.534 (χ²)
LOS (median (IQR))	5.00 (3.00, 9.00)	6.00 (3.00, 10.00)	<0.001	-5.902 (Z)
Lab values				
Pro-BNP (median (IQR))	3,926.50 (1,460.00, 9,734.00)	3471.00 (1,306.00, 9,007.00)	0.044	2.009 (Z)
WBC (median (IQR))	8.84 (6.73, 11.85)	9.49 (6.87, 13.04)	<0.001	-4.996 (Z)
Lactate (median (IQR))	1.60 (1.20, 2.30)	1.60 (1.20, 2.20)	0.725	0.352 (Z)
Hemoglobin (median (IQR))	11.40 [9.80, 13.00]	11.00 [9.40, 12.50]	<0.001	8.641 (Z)
Sodium (median (IQR))	138.00 [135.00, 140.00]	137.00 [134.00, 140.00]	<0.001	5.748 (Z)
CRP > 10 mg/dL (%)	15,824 (19.9%)	520 (29.9%)	<0.001	106.955 (χ²)
Troponin I (median (IQR))	0.06 (0.03, 0.20)	0.05 (0.03, 0.13)	<0.001	3.803 (Z)
Medication use				
cDMARDs	593 (0.7%)	303 (17%)	<0.001	4,344.376 (χ²)
bDMARDs	67 (<0.1%)	78 (4.5%)	<0.001	1,827.363 (χ²)
sDMARDs	3 (<0.1%)	12 (0.7%)	<0.001	N/A (Fisher’s)
Steroids	3,552 (4.5%)	384 (22.1%)	<0.001	1,146.854 (χ²)
Phenotype				
HFpEF	36,955 (46%)	970 (56%)		77.391 (χ²)
HFrEF	26,138 (33%)	409 (24%)		
Unspecified	16,441 (21%)	355 (20%)		

Following PSM, patient baseline characteristics and comorbidities were well balanced between the non-RA and RA groups, with absolute SMD < 0.1 for all matched variables (Supplementary materials 3 and 4).

**Figure 1:**
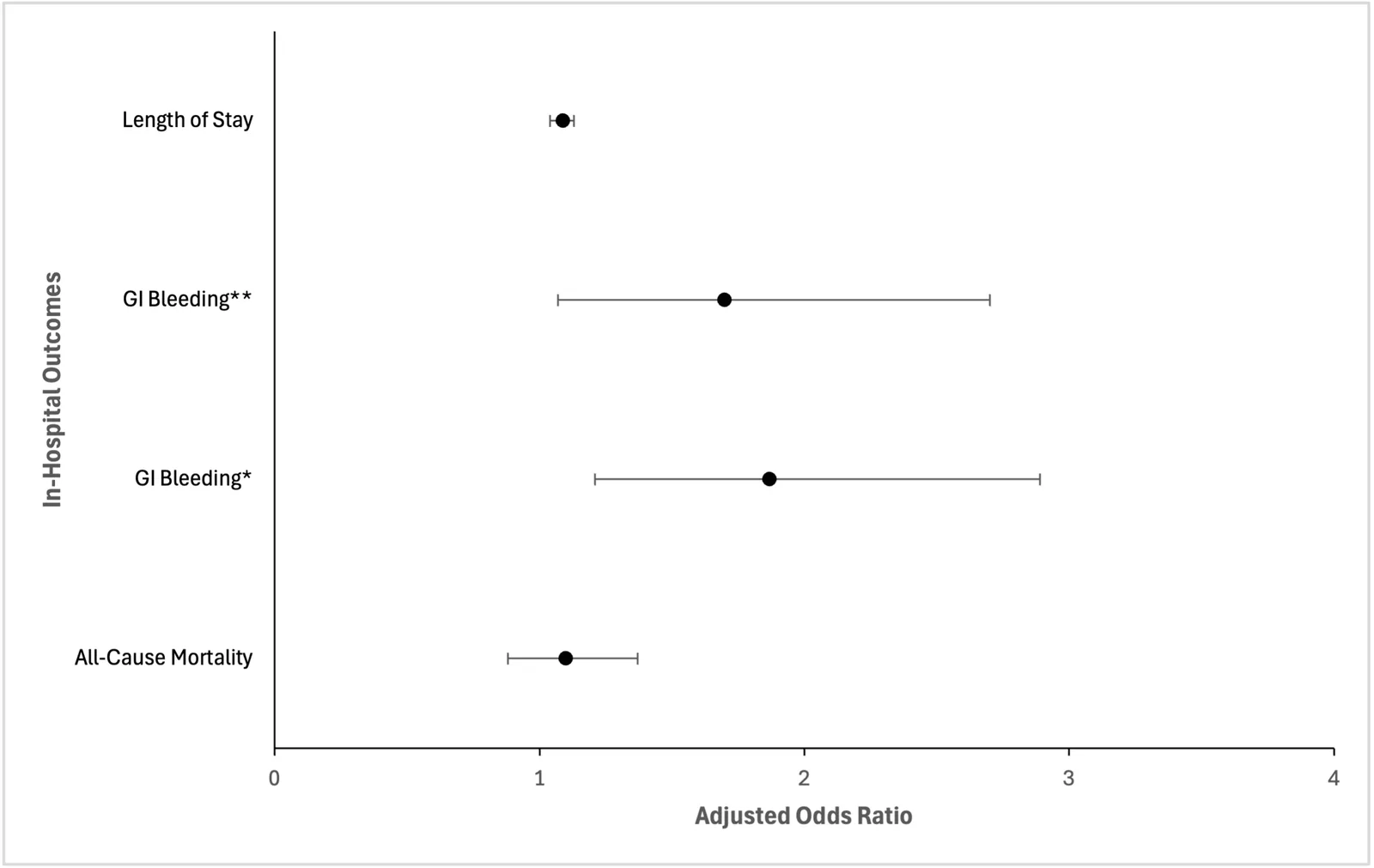
Adjusted odds ratios for mortality, LOS, and GI bleeding in HF patients with RA ^*^Without adjustment of in-hospital NSAID, corticosteroid, anticoagulation, or antiplatelet use. ^**^With adjustment of in-hospital NSAID, corticosteroid, anticoagulation, or antiplatelet use. LOS: length of stay, GI: gastrointestinal, HF: heart failure, NSAID: nonsteroidal anti-inflammatory drug.

**Figure 2:**
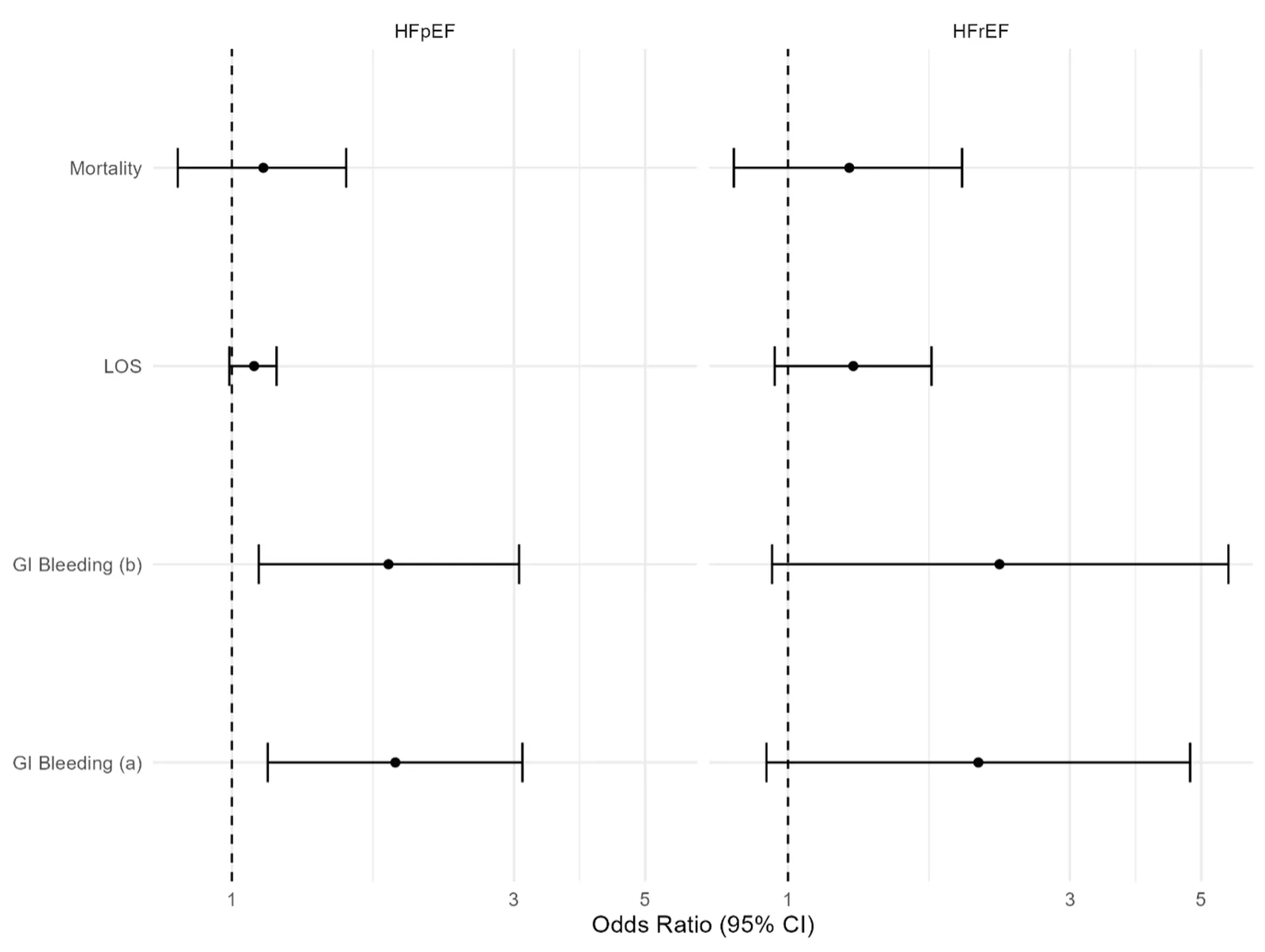
Adjusted odds ratios for mortality, LOS, and GI bleeding in RA patients stratified by HFpEF versus HFrEF ^a^Without adjustment of in-hospital NSAID, corticosteroid, anticoagulation, or antiplatelet use. ^b^With adjustment of in-hospital NSAID, corticosteroid, anticoagulation, or antiplatelet use. LOS: length of stay, GI: gastrointestinal, RA: rheumatoid arthritis, HFpEF: heart failure with preserved ejection fraction, HFrEF: heart failure with reduced ejection fraction, NSAID: nonsteroidal anti-inflammatory drug.

Adjusted analyses controlling for confounders including sex, age, ethnicity, comorbidities, and lab values are presented in Table 2. Patients with RA demonstrated greater odds of GI bleeding (OR 1.61; 95% CI 1.04-2.48) and longer LOS (OR 1.17; 95% CI 1.04-1.32). The effect of RA on GI bleeding was attenuated and no longer statistically significant after further adjustment for in-hospital medication use including anticoagulants, antiplatelets, NSAIDs, and corticosteroids (OR 1.48; 95% CI 0.94-2.33) (Figure 1). HF patients with RA did not reveal greater odds of all-cause mortality (OR 1.11; 95% CI 0.88-1.40). Further stratified analysis by HF phenotype (HFpEF vs. HFrEF) demonstrated consistent trends for mortality, LOS, and GI bleeding between the two subgroups, while the HFpEF group had narrower confidence intervals (Figure 2).

**Table 2:** Multivariable regression analysis of HF patients with RA and mortality, GI bleeding, and hospital length of stay ^a^Without adjustment of NSAID, corticosteroid, anticoagulation, or antiplatelet use. ^b^With adjustment of NSAID, corticosteroid, anticoagulation, or antiplatelet use. HF: heart failure, RA: rheumatoid arthritis. GI: gastrointestinal, CAD: coronary artery disease, COPD: chronic obstructive pulmonary disease, CKD: chronic kidney disease, PAD: peripheral artery disease, SLE: systemic lupus erythematosus, TCP: thrombocytopenia, WBC: white blood cell, NSAIDs: nonsteroidal anti-inflammatory drugs.

	All-cause mortality			GI bleeding^a^			GI bleeding^b^			Length of stay		
Variables	aOR (95% CI)	z-statistic	p-value	aOR (95% CI)	z-statistic	p-value	aOR (95% CI)	z-statistic	p-value	aOR (95% CI)	z-statistic	p-value
RA	1.11 (0.88-1.40)	0.91	0.363	1.61 (1.04-2.48)	2.13	0.033	1.48 (0.94-2.33)	1.68	0.092	1.17 (1.04-1.32)	2.64	0.008
Hyperlipidemia	0.82 (0.65-1.04)	-1.62	0.106	1.10 (0.69-1.75)	0.39	0.695	1.12 (0.70-1.78)	0.48	0.634	1.06 (0.97-1.15)	1.25	0.212
CAD	0.83 (0.65-1.06)	-1.49	0.137	0.96 (0.57-1.62)	-0.15	0.884	0.97 (0.58-1.63)	-0.11	0.912	0.96 (0.87-1.06)	-0.73	0.463
Hypertension	0.75 (0.50-1.13)	-1.38	0.168	0.67 (0.29-1.56)	-0.93	0.354	0.65 (0.28-1.51)	-1.00	0.316	1.03 (0.89-1.19)	0.35	0.728
COPD	1.21 (0.93-1.57)	1.45	0.148	1.08 (0.59-1.97)	0.24	0.807	1.01 (0.54-1.87)	0.02	0.987	1.02 (0.94-1.12)	0.55	0.584
Diabetes	0.76 (0.61-0.96)	-2.29	0.022	1.09 (0.65-1.84)	0.33	0.741	1.12 (0.67-1.90)	0.44	0.661	0.99 (0.91-1.07)	-0.33	0.741
Tobacco use	1.06 (0.83-1.35)	0.46	0.649	1.07 (0.65-1.77)	0.26	0.798	1.05 (0.64-1.72)	0.18	0.855	1.09 (0.97-1.21)	1.44	0.149
CKD	1.00 (0.78-1.28)	0.01	0.991	1.10 (0.64-1.87)	0.34	0.735	1.14 (0.66-1.95)	0.47	0.64	1.00 (0.92-1.09)	0.07	0.943
Obesity	0.96 (0.72-1.28)	-0.28	0.782	1.09 (0.63-1.88)	0.31	0.754	1.05 (0.60-1.82)	0.16	0.871	1.11 (1.03-1.20)	2.78	0.006
PAD	1.21 (0.75-1.93)	0.78	0.434	1.34 (0.54-3.30)	0.64	0.524	1.30 (0.53-3.19)	0.58	0.56	1.27 (1.09-1.48)	3.09	0.002
SLE	1.80 (1.14-2.83)	2.54	0.011	0.92 (0.33-2.55)	-0.17	0.868	0.83 (0.29-2.38)	-0.34	0.733	1.01 (0.88-1.15)	0.07	0.943
TCP	1.78 (1.30-2.44)	3.61	<0.001	2.30 (1.33-3.98)	2.97	0.003	2.26 (1.31-3.90)	2.94	0.003	1.60 (1.43-1.80)	7.97	<0.001
Race/ethnicity												
Black	1.06 (0.63-1.79)	0.21	0.836	1.75 (0.72-4.23)	1.24	0.215	1.82 (0.76-4.38)	1.34	0.18	1.05 (0.88-1.26)	0.56	0.574
Hispanic	1.18 (0.88-1.59)	1.11	0.268	0.93 (0.49-1.78)	-0.21	0.835	0.91 (0.48-1.72)	-0.28	0.782	1.09 (0.99-1.19)	1.83	0.068
Other	0.98 (0.55-1.74)	-0.08	0.933	1.32 (0.47-3.73)	0.52	0.603	1.32 (0.47-3.73)	0.53	0.597	1.09 (0.93-1.27)	1.05	0.294
Age	1.03 (1.02 - 1.04)	4.73	<0.001	1.00 (0.98 - 1.02)	0.31	0.759	1.00 (0.98 - 1.03)	0.44	0.657	0.99 (0.99 - 1.00)	-4.16	<0.001
Gender												
Male	1.08 (0.82-1.43)	0.55	0.58	0.97 (0.55-1.72)	-0.11	0.91	0.98 (0.56-1.74)	-0.05	0.957	0.96 (0.88-1.06)	-0.75	0.454
Pro-BNP	1.21 (1.10-1.33)	3.81	<0.001	1.08 (0.90-1.29)	0.83	0.408	1.10 (0.92-1.31)	1.03	0.304	1.02 (0.99-1.05)	1.14	0.254
WBC	1.39 (1.11-1.75)	2.88	0.004	0.92 (0.61-1.37)	-0.43	0.665	0.88 (0.59-1.31)	-0.61	0.542	1.08 (1.01-1.16)	2.12	0.034
Lactic acid	3.08 (2.55-3.72)	11.65	<0.001	2.00 (1.31-3.06)	3.23	0.001	2.04 (1.32-3.13)	3.24	0.001	1.01 (0.94-1.09)	0.24	0.807
Sodium	1.00 (0.97-1.02)	-0.28	0.776	0.97 (0.92-1.02)	-1.13	0.26	0.97 (0.92-1.02)	-1.11	0.266	0.99 (0.99-1.00)	-1.82	0.069
Hemoglobin	0.95 (0.90-1.00)	-2.09	0.037	0.84 (0.75-0.94)	-3.05	0.002	0.83 (0.74-0.94)	-3.08	0.002	0.98 (0.96-0.99)	-2.67	0.008
Troponin I	1.26 (1.15-1.38)	4.83	<0.001	1.10 (0.90-1.35)	0.93	0.352	1.11 (0.90-1.36)	0.99	0.324	1.04 (0.99-1.11)	1.46	0.144
NSAIDs							4.68 (1.82-12.03)	3.21	0.001			
Corticosteroids							1.46 (0.89-2.39)	1.50	0.134			
Antiplatelets							1.03 (0.54-1.98)	0.10	0.922			
Anticoagulant							1.15 (0.62-2.12)	0.45	0.652			

Among HF patients with RA, higher CRP levels were associated with a greater risk of all outcomes except pneumonia. The use of cDMARDs prior to admission was associated with a higher risk of cardiac arrest, MACE, and MI/ACS. bDMARDs were associated with a greatly increased risk of MI/ACS (aOR 9.03), but a lower risk of stroke and atrial fibrillation. Pre-hospital use of corticosteroids was associated with greater odds of in-hospital VTE and stroke (Table 3). This subgroup analysis was fitted for exploratory hypothesis generation, not for strict inference or prediction. So, the ORs should be interpreted with caution.

**Table 3:** Multivariable Firth’s penalized logistic regression analysis on cardiovascular outcomes among HF patients with RA Models were adjusted for age, gender, race/ethnicity, comorbidities, and laboratory biomarkers. DMARDs and corticosteroids are recorded medical use at home before hospital admission. VTE: venous thromboembolism, MACE:  major adverse cardiac events, MI/ACS: myocardial infarction/acute coronary syndrome, HF: heart failure, RA: rheumatoid arthritis, DMARDs: disease-modifying antirheumatic drugs.

	CRP			cDMARDs			bDMARDs			Corticosteroids		
Variables	aOR (95% CI)	z- statistic	p-value	aOR (95% CI)	z- statistic	p-value	aOR (95% CI)	z- statistic	p-value	aOR (95% CI)	z- statistic	p-value
VTE	4.21 (2.48-7.14)	5.33	<0.001	0.52 (0.26-1.04)	-1.84	0.066	0.81 (0.23-2.82)	-0.33	0.739	2.46 (1.38-4.37)	3.06	0.002
Cardiogenic shock	4.02 (2.14-7.55)	4.34	<0.001	1.01 (0.55-1.86)	0.03	0.977	2.66 (1.01-6.98)	1.98	0.047	0.90 (0.44-1.84)	-0.30	0.767
Stroke	2.03 (1.10-3.74)	2.26	0.024	1.45 (0.75-2.79)	1.10	0.272	0.48 (0.26-0.87)	-2.41	0.016	2.01 (1.11-3.65)	2.29	0.022
Atrial fibrillation	3.38 (1.88-6.09)	4.07	<0.001	0.83 (0.40-1.71)	-0.52	0.606	0.36 (0.17-0.78)	-2.58	0.01	0.46 (0.20-1.01)	-1.93	0.053
Cardiac arrest	2.54 (1.40-4.61)	3.08	0.002	2.61 (1.36-5.03)	2.87	0.004	2.74 (1.03-7.31)	2.02	0.044	0.62 (0.32-1.21)	-1.40	0.16
MACE	2.89 (1.77-4.71)	4.25	<0.001	2.06 (1.19-3.57)	2.57	0.01	1.93 (0.85-4.41)	1.56	0.119	0.73 (0.41-1.32)	-1.03	0.305
MI/AC	2.71 (1.31-5.64)	2.67	0.007	7.61 (3.50-16.53)	5.13	<0.001	9.03 (2.72-29.90)	3.60	<0.001	0.62 (0.26-1.47)	-1.09	0.275

## Discussion

In this large retrospective cohort of patients hospitalized with HF, concomitant RA was associated with prolonged hospitalizations as well as a higher risk of GI bleeding complications. However, in-hospital mortality or cardiovascular events were not associated with concomitant RA. Among patients with RA, elevated CRP levels along with the use of DMARDs or corticosteroids were independently associated with a higher risk of thromboembolic and cardiac complications.

Previous studies have suggested that systemic inflammation in RA may exacerbate atherosclerosis and HF progression, resulting in adverse long-term outcomes [7,15]. Systemic inflammation is a key determinant and harbinger of adverse cardiovascular events in RA, with cardiovascular disease as the most common cause of premature death [16]. Patients with RA carry a 1.5- to 2-fold greater risk of CAD and MI compared to the general population [17]. Chronically elevated acute-phase reactants and proinflammatory cytokines may promote atherogenesis, endothelial dysfunction, and ultimately acute coronary syndrome (ACS) in these patients [18]. Furthermore, emerging evidence suggests there may be a role for the use of inflammatory cytokines and novel biomarkers to become integrated into current risk models for the prognostication of ACS, such as the GRACE score [19]. These inflammatory mechanisms also exacerbate HF progression, resulting in adverse long-term outcomes. Our finding of similar in-hospital mortality rates among patients with and without RA may reflect the evolving advances in both rheumatologic and cardiovascular standard of care. Over the past decade, the widespread adoption of DMARDs and other biologic agents, for example, has led to improved control of systemic inflammation and reduced cardiovascular comorbidity among RA populations. Concurrently, advances in inpatient HF management, including standardized guideline-directed medical therapy options and early specialist involvement, have enhanced short-term survival across diverse patient subgroups [7,20]. Together, these developments may have mitigated the acute mortality risk when RA patients are hospitalized for HF, even if RA continues to influence HF disease trajectory and post-discharge outcomes [11]. Our findings may reflect a transition period in which advances in disease control and modern inpatient care have attenuated the historically elevated short-term mortality risk associated with systemic inflammation.

Nevertheless, recent nationwide analyses have also shown contrasting findings. A retrospective cohort using the National Readmission Database found that patients with RA readmitted within 90 days following an index HF hospitalization had significantly higher in-hospital mortality and readmission risk compared to patients who did not have RA [10]. In contrast, our study demonstrated that RA status was not associated with adverse prognosis during the index HF hospitalization, as no significant differences in in-hospital mortality or cardiovascular complications were observed. This difference may reflect variation in study design and the timing of outcome assessment; our study examined only acute inpatient outcomes, whereas the cohort by Chandrupatla et al. [10] evaluated readmissions and post-discharge mortality, where the persistent effects of systemic inflammation and comorbid disease may have continued to increase the overall risk of adverse outcomes. Another study utilizing the Swedish HF Registry examined the risk of incident HF as well as long-term cardiovascular complications and mortality up to five years following initial HF diagnosis. The investigators found an increased long-term mortality risk among patients with RA compared to those without it; however, their analysis did not evaluate complications in the acute inpatient setting [21].

The higher incidence of GI and major bleeding among RA patients is consistent with the combined effects of chronic corticosteroid use, NSAID exposure, and vascular fragility associated with long-standing inflammation [16]. This bleeding risk may also be compounded by initiation of DVT prophylaxis in the inpatient setting, particularly among patients with inflammatory arthritis due to their elevated baseline risk of VTE [22,23]. Notably, this analysis did not account for anticoagulant or antiplatelet exposure, which represents an important unmeasured confounder. These findings highlight the need for targeted strategies for the prevention of major and GI bleeding risk in this patient population, including early risk stratification, careful review of concomitant anticoagulation and antiplatelet therapies, and avoidance of unnecessary NSAID and corticosteroid exposure. Proactive implementation of these measures may mitigate bleeding events while preserving the benefits of thromboprophylaxis in this high-risk subgroup.

Several mechanisms likely explain the bleeding risk observed in RA patients with HF. Chronic NSAID and steroid use in this population may precipitate direct GI mucosal injury and a greater likelihood of peptic ulcer formation. Moreover, the presence of extra-articular manifestations in RA patients has been associated with more serious GI bleeding events, which may be related to disease severity contributing to intestinal vulnerability [12]. Endothelial dysfunction driven by chronic inflammation, oxidative stress, and circulating autoantibodies and immune complexes may also compromise vascular integrity and increase susceptibility to bleeding in these patients [13]. Our cohort also demonstrated a greater tendency for RA patients to have thrombocytopenia, which also places them at greater risk of developing bleeding events during hospital admission in the setting of HF decompensation. Platelets in RA circulate in a chronically activated state that may contribute to hemorrhagic complications via consumptive mechanisms [14]. Lastly, initiation of pharmacologic VTE prophylaxis during HF hospitalization may also significantly compound bleeding risk in these patients.

Longer hospital stays are consistent with prior reports [24,25] and may reflect the complexity of managing RA comorbidities, polypharmacy, and higher baseline frailty. This may also suggest delayed recovery and increased need for multidisciplinary coordination. Elevated CRP as a marker of active systemic inflammation was also associated with a higher risk of MACE and VTE, consistent with the central role of inflammation in increasing the risk of thrombotic and cardiovascular complications among these patients. The association between DMARDs and adverse cardiovascular outcomes should be interpreted with caution given the potential for confounding by indication. Patients with more severe or refractory RA and greater systemic inflammatory burden are more likely to receive DMARDs and biologic agents. Without stratification by specific DMARD class or RA disease severity scores, it is not possible to distinguish the effects of disease severity and DMARD therapy individually in this cohort. The heterogeneous effects of various DMARD classes on cardiovascular outcomes further underscore the important differences in cardiovascular risk profiles among individual agents [26,27]. 

Our findings underscore the importance of careful inpatient monitoring of bleeding and thrombotic risks among HF patients with RA, particularly those receiving DMARDs or corticosteroids. In our cohort, DMARD use was associated with higher odds of VTE, cardiac arrest, MACE, and MI/ACS, suggesting that RA patients requiring these therapies represent a uniquely vulnerable subgroup. Although methotrexate has well-described cardioprotective effects, including reductions in cardiovascular mortality risk and MI incidence [28,29], the impact of other cDMARDs remains less certain. Observational studies have shown mixed or inconclusive results particularly with sulfasalazine, leflunomide, and hydroxychloroquine, with some agents even linked to worsened cardiac remodeling, MI, and stroke [27,30-32]. bDMARDs also vary, with agents such as TNF inhibitors and abatacept showing an association with lower MACE risk [26], whereas IL-12/23 and JAK2 inhibitors carry substantially higher risk of MACE and VTE [33]. Thus, the association between DMARD use and worse cardiovascular outcomes in our cohort may reflect either more severe RA with higher inflammatory burden or direct medication-related toxicity, consistent with the elevated risks of MACE and VTE reported with certain DMARD classes. However, our analysis did not stratify outcomes by specific DMARD subtype, which limits our ability to determine whether particular agents disproportionately contributed to these findings. In the case of amplified inflammatory status, this may worsen endothelial dysfunction, increase plaque instability, and promote thrombosis in RA patients during acute HF decompensation, contributing to the complications observed. These findings reinforce the need for close inpatient surveillance, including vigilant assessment for evolving VTE or GI bleeding and optimization of prophylaxis and medication management upon admission.

Several limitations of this study should be considered when interpreting these findings. While our data from a large tertiary urban healthcare system afforded a proportionately greater number of patients and increased the validity of our findings, the retrospective observational design precludes the establishment of causal relationships between RA and inpatient outcomes among hospitalized HF patients. Although multivariate adjustment and PSM were applied to minimize confounding, residual confounding from other unmeasured variables including disease duration, RA activity scores, HF severity, outpatient medication adherence, or timing of DMARD or corticosteroid initiation could have influenced our findings. Notably, data on anticoagulant, antiplatelet, and aspirin exposure were not systematically captured in this dataset and represent a significant unmeasured confounder in the bleeding analysis. Furthermore, our analysis did not stratify by HF phenotype (HFrEF vs. HFpEF), and inability to capture severity classification (e.g., NYHA class) is an important limitation, as these factors strongly influence inpatient outcomes. The aggregate DMARD variable used in this analysis also did not distinguish between conventional, biologic, and targeted synthetic agents, which may have heterogeneous cardiovascular risk profiles. Moreover, the use of administrative ICD-10 coding to identify diagnoses may introduce misclassification bias due to variation in coding accuracy across the encounters. This analysis also focused exclusively on in-hospital outcomes, and thus, long-term prognostic implications like readmission and post-discharge mortality were beyond the scope of this study. Lastly, because this study was conducted in a single large healthcare system, generalizability to other populations or healthcare settings may be limited.

## Conclusions

In this large, contemporary cohort of patients hospitalized with HF, concomitant RA was associated with a greater risk of GI and major bleeding, along with prolonged hospitalization, but not with increased in-hospital mortality or cardiovascular events independently. Among RA patients with HF, those with high CRP levels and on DMARD therapy demonstrated an association with VTE, cardiac arrest, MACE, and MI/ACS, suggesting a possible relationship between systemic inflammation, disease severity, and inpatient outcomes. These findings highlight the importance of vigilant inpatient monitoring, along with careful review of home medications that may increase bleeding risk in the setting of HF decompensation. Future studies should evaluate the cardiovascular and thrombotic associations of specific DMARD classes, including conventional synthetic and biologic agents, while controlling for RA disease severity and antithrombotic medication exposure as potential confounders, since greater inflammatory activity may be associated with worse outcomes during HF hospitalizations. 

## Appendices

Supplementary material 1

**Table 4:** ICD-10 codes for baseline comorbidities

Baseline comorbidities	
Variables	ICD-10 codes
Hypertension	I10, I11.0, I11.9, I12.0, I12.9, I13.0, I13.10, I13.11, I13.2, I15.0, I15.1, I15.2, I15.8, I15.9
Diabetes mellitus	E08.0, E08.1, E08.2, E08.3, E08.4, E08.5, E08.6, E08.8, E08.9, E09.0, E09.1, E09.2, E09.3, E09.4, E09.5, E09.6, E09.8, E09.9, E10.0, E10.1, E10.2, E10.3, E10.4, E10.5, E10.6, E10.8, E10.9, E11.0, E11.1, E11.2, E11.3, E11.4, E11.5, E11.6, E11.8, E11.9, E13.0, E13.1, E13.2, E13.3, E13.4, E13.5, E13.6, E13.8, E13.9
Tobacco use	Z72.0, Z87.891, F17.200, F17.201, F17.210, F17.211, F17.213, F17.218, F17.219, F17.220, F17.221, F17.223, F17.228, F17.229, F17.290, F17.291, F17.293, F17.298, F17.299
Thrombocytopenia	D69.1, D69.3, D69.41, D69.42, D69.49, D69.51, D69.52, D69.59, D69.6
Obesity	E66.0, E66.01, E66.09, E66.1, E66.2, E66.3, E66.8, E66.9
Hyperlipidemia	E78.0, E78.1, E78.2, E78.3, E78.4, E78.5, E78.6, E78.70, E78.71, E78.72, E78.79, E78.81, E78.89, E78.9t
Coronary artery disease	I25.10, I25.11, I25.118, I25.119, I25.2, I25.5, I25.6, I25.700, I25.701, I25.708, I25.709, I25.710, I25.711, I25.718, I25.719, I25.720, I25.721, I25.728, I25.729, I25.730, I25.731, I25.738, I25.739, I25.750, I25.751, I25.758, I25.759, I25.760, I25.761, I25.768, I25.769, I25.790, I25.791, I25.798, I25.799, I25.810, I25.811, I25.812, I25.818, I25.82, I25.83, I25.84, I25.89, I25.9
Peripheral artery disease	I70.200, I70.201, I70.202, I70.203, I70.208, I70.209, I70.210, I70.211, I70.212, I70.213, I70.218, I70.219, I70.220, I70.221, I70.222, I70.223, I70.228, I70.229, I70.230, I70.231, I70.232, I70.233, I70.238, I70.239, I70.240, I70.241, I70.242, I70.243, I70.248, I70.249, I70.25, I70.26, I70.261, I70.262, I70.263, I70.268, I70.269, I70.270, I70.271, I70.272, I70.273, I70.278, I70.279, I70.290, I70.291, I70.292, I70.293, I70.298, I70.299, I70.30, I70.31, I70.32, I70.33, I70.34, I70.35, I70.36, I70.37, I70.38, I70.39, I73.9
Chronic obstructive pulmonary disease	J44.0, J44.1, J44.9
Chronic kidney disease	N18.1, N18.2, N18.30, N18.31, N18.32, N18.4, N18.5, N18.6, N18.9
Systemic lupus erythematosus	M32.0, M32.1, M32.10, M32.11, M32.12, M32.13, M32.14, M32.15, M32.19, M32.8, M32.9

Supplementary material 2 

**Table 5:** ICD-10 codes for outcomes

In-hospital outcomes	
Variables	ICD-10 codes
Myocardial infarction (MI)/acute coronary syndrome (ACS)	I20.0, I21.0, I21.01, I21.02, I21.09, I21.1, I21.11, I21.19, I21.2, I21.21, I21.29, I21.3, I21.4, I21.9, I22.0, I22.1, I22.2, I22.8, I22.9, I24.0, I24.1, I24.8, I24.9
Cardiac arrest	I46.2, I46.8, I46.9
Cardiogenic shock	R57.0
Stroke, ischemic stroke, transient ischemic attack (TIA), hemorrhagic stroke	I63.0, I63.1, I63.2, I63.3, I63.4, I63.5, I63.6, I63.8, I63.9, G45.0, G45.1, G45.2, G45.3, G45.4, G45.8, G45.9 I60.0, I60.1, I60.2, I60.3, I60.4, I60.5, I60.6, I60.7, I60.8, I60.9, I61.0, I61.1, I61.2, I61.3, I61.4, I61.5, I61.6, I61.8, I61.9, I62.0, I62.1, I62.9
Atrial fibrillation	I48.0, I48.11, I48.19, I48.20, I48.21, I48.91
Ventricular arrhythmias	I47.0, I47.2, I49.01, I49.02, I49.09
Valvulopathy	I34.0, I34.1, I34.8, I34.9, I35.0, I35.1, I35.2, I35.8, I35.9, I36.0, I36.1, I36.2, I36.8, I36.9, I37.0, I37.1, I37.2, I37.8, I37.9, I51.1, I51.2
Major adverse cardiac events (defined as any of the following: MI/ACS, cardiac arrest, cardiogenic shock, or stroke)	I20.0, I21.0, I21.01, I21.02, I21.09, I21.1, I21.11, I21.19, I21.2, I21.21, I21.29, I21.3, I21.4, I21.9, I22.0, I22.1, I22.2, I22.8, I22.9, I24.0, I24.1, I24.8, I24.9 I46.2, I46.8, I46.9 R57.0 I63.0, I63.1, I63.2, I63.3, I63.4, I63.5, I63.6, I63.8, I63.9, G45.0, G45.1, G45.2, G45.3, G45.4, G45.8, G45.9, I60.0, I60.1, I60.2, I60.3, I60.4, I60.5, I60.6, I60.7, I60.8, I60.9, I61.0, I61.1, I61.2, I61.3, I61.4, I61.5, I61.6, I61.8, I61.9, I62.0, I62.1, I62.9
Respiratory failure	J96.00, J96.01, J96.02, J96.10, J96.11, J96.12, J96.20, J96.21, J96.22, J96.90, J96.91, J96.92, J80
Pneumonia	J18.9, J15.9, J15.0, J15.1, J15.2, J15.3, J15.4, J15.5, J15.6, J15.7, J15.8, J16.0, J16.8
Sepsis	A40.0, A40.1, A40.2, A40.3, A40.8, A40.9, A41.0, A41.1, A41.2, A41.3, A41.4, A41.50, A41.51, A41.52, A41.53, A41.59, A41.81, A41.89, A41.9, R65.20, R65.21, R57.2
Any major bleeding	I60.0, I60.1, I60.2, I60.3, I60.4, I60.5, I60.6, I60.7, I60.8, I60.9, I61.0, I61.1, I61.2, I61.3, I61.4, I61.5, I61.6, I61.8, I61.9, I62.0, I62.1, I62.9, K25.0, K25.2, K25.4, K25.6, K26.0, K26.2, K26.4, K26.6, K27.0, K27.2, K27.4, K27.6, K28.0, K28.2, K28.4, K28.6, K29.01, K29.21, K29.31, K29.41, K29.51, K29.61, K29.71, K29.81, K29.91, K62.5, K92.0, K92.1, K92.2, N02.0, N02.1, N02.2, N02.3, N02.4, N02.5, N02.6, N02.8, N02.9, N30.01, N30.11, N30.21, N30.31, N30.41, N30.81, N30.91, N32.89, N36.8, N39.0, R31.0, R31.1, R31.9, R04.0, R04.1, R04.2, R04.8, R04.9, H11.3, H35.6
GI bleeding	K25.0, K25.2, K25.4, K25.6, K26.0, K26.2, K26.4, K26.6, K27.0, K27.2, K27.4, K27.6, K28.0, K28.2, K28.4, K28.6, K29.01, K29.21, K29.31, K29.41, K29.51, K29.61, K29.71, K29.81, K29.91, K62.5, K92.0, K92.1, K92.2
Acute kidney injury (AKI)	N17.0, N17.1, N17.2, N17.8, N17.9
Venous thromboembolism (VTE)	I26.0, I26.01, I26.02, I26.09, I26.9, I26.90, I26.92, I26.93, I26.94, I26.99, I82.4, I82.40, I82.401, I82.402, I82.403, I82.409, I82.41, I82.411, I82.412, I82.413, I82.419, I82.42, I82.421, I82.422, I82.423, I82.429, I82.43, I82.431, I82.432, I82.433, I82.439, I82.44, I82.441, I82.442, I82.443, I82.449, I82.49, I82.5, I82.50, I82.501, I82.502, I82.503, I82.509, I82.51, I82.511, I82.512, I82.513, I82.519, I82.52, I82.521, I82.522, I82.523, I82.529, I82.529, I82.53, I82.531, I82.532, I82.533, I82.539, I82.54, I82.541, I82.542, I82.543, I82.549, I82.59, I82.6, I82.61, I82.62, I82.62, I82.63, I82.69, I82.8, I82.81, I82.82, I82.83, I82.89, I82.9

Supplementary material 3 

**Table 6:** Balance check following propensity score matching

Variable	Level	Non-RA	RA	SMD
Age		81.0 (73.0, 87.0)	80.0 (74.0, 86.0)	-0.016
Hyperlipidemia	Yes	3,112 (59.6%)	1,037 (59.6%)	-0.026
CAD	Yes	2,406 (46.1%)	810 (46.6%)	0.005
Hypertension	Yes	4,915 (94.2%)	1,621 (93.2%)	-0.017
COPD	Yes	1,196 (22.9%)	421 (24.2%)	0.029
Diabetes	Yes	2,396 (45.9%)	807 (46.4%)	-0.015
Tobacco use	Yes	2,143 (41.1%)	742 (42.6%)	0.035
CKD	Yes	1,775 (34.0%)	620 (35.6%)	0.055
Obesity	Yes	1,375 (26.3%)	484 (27.8%)	0.031
PAD	Yes	251 (4.8%)	105 (6.0%)	0.058
SLE	Yes	238 (4.6%)	79 (4.5%)	-0.003
TCP	Yes	608 (11.6%)	222 (12.8%)	0.022
Sex	Male	1,290 (24.7%)	431 (24.8%)	-0.000
Ethnicity	Black	390 (7.5%)	140 (8.1%)	0.019
	Hispanic	3,615 (69.3%)	1,177 (67.7%)	-0.030
	Other	240 (4.6%)	93 (5.3%)	0.027
	White	975 (18.7%)	328 (18.9%)	0.007

Supplementary material 4 

**Table 7:** Regression analysis on outcomes among patients with HF and RA

Outcomes	Normal CRP	High CRP	DMARDS	Corticosteroids
Respiratory failure	1.89 (0.60-5.94)	2.92 (1.96-4.37)	1.38 (0.84-2.26)	1.29 (0.77-2.15)
Mortality	0.96 (0.29-3.14)	2.20 (1.53-3.17)	0.93 (0.58-1.51)	0.92 (0.58-1.45)
VTE	1.08 (0.53-2.18)	4.22 (2.42-7.34)	0.38 (0.19-0.77)	2.91 (1.61-5.26)
GI bleeding	2.55 (0.69-9.44)	2.39 (1.34-4.28)	1.39 (0.74-2.60)	0.88 (0.48-1.63)
Major bleeding	1.80 (0.65-4.99)	2.14 (1.42-3.22)	1.09 (0.66-1.80)	1.07 (0.64-1.77)
Pneumonia	3.55 (1.24-10.17)	1.66 (0.97-2.84)	0.99 (0.57-1.75)	1.02 (0.56-1.88)
Ventricular arrhythmias	3.98 (1.18-13.36)	2.80 (1.60-4.92)	0.64 (0.30-1.39)	0.65 (0.30-1.43)
Sepsis	3.85 (1.24-11.96)	5.56 (3.27-9.47)	1.28 (0.69-2.36)	1.02 (0.57-1.84)
AKI	0.83 (0.25-2.74)	1.98 (1.33-2.94)	0.95 (0.58-1.55)	1.25 (0.79-1.99)
Cardiogenic shock	6.68 (1.70-26.20)	3.90 (2.03-7.50)	0.85 (0.44-1.65)	0.96 (0.45-2.05)
Stroke	5.77 (1.87-17.82)	2.02 (1.09-3.76)	1.07 (0.55-2.08)	1.93 (1.03-3.62)
Atrial fibrillation	2.52 (0.69-9.20)	3.56 (1.97-6.45)	0.62 (0.29-1.32)	0.51 (0.22-1.14)
Cardiac arrest	4.84 (1.41-16.60)	2.62 (1.43-4.83)	2.32 (1.20-4.48)	0.65 (0.33-1.29)
MACE	4.69 (1.59-13.87)	2.95 (1.76-4.94)	1.85 (1.07-3.19)	0.78 (0.42-1.44)
MI/ACS	1.69 (0.29-9.72)	3.83 (1.69-8.70)	9.87 (4.09-23.84)	0.78 (0.31-1.98)
LOS	1.49 (1.24-1.78)	1.64 (1.51-1.78)	0.98 (0.90-1.07)	1.07 (0.98-1.16)
